# Tocilizumab in COVID-19: Factors Associated With Mortality Before and After Treatment

**DOI:** 10.3389/fphar.2021.620187

**Published:** 2021-07-01

**Authors:** Luis Sarabia De Ardanaz, Jose M. Andreu-Ubero, Miriam Navidad-Fuentes, Miguel Ángel Ferrer-González, Victor Ruíz del Valle, Inmaculada Salcedo-Bellido, Rocío Barrios-Rodríguez, Rafael Cáliz-Cáliz, Pilar Requena

**Affiliations:** ^1^ Departamento de Reumatología, Hospital Universitario Virgen de las Nieves, Granada, Spain; ^2^ Departamento de Medicina Preventiva y Salud Pública, Universidad de Granada, Granada, España; ^3^ Instituto de Investigación Biosanitaria (ibs.Granada), Granada, Spain; ^4^ Consortium for Biomedical Research in Epidemiology and Public Health (CIBERESP), Madrid, Spain

**Keywords:** COVID-19, immunosupression, tocilizumab, mortality, risk factor, platelet

## Abstract

Tocilizumab (TCZ) has been administered in SARS-CoV-2 pneumonia but the factors associated with mortality before and after treatment remain unclear. Cox regression models were used to estimate the predictors of time to death in a cohort of hospitalized patients with COVID-19 receiving TCZ. In addition, the mean differences between discharged and deceased patients in laboratory parameters measured before and 3, 6 and 9 days after TCZ administration were estimated with weighted generalized estimation equations. The variables associated with time to death were immunosuppression (Hazard Ratio-HR 3.15; 95% confidence interval-CI 1.17, 8.51), diabetes mellitus (HR 2.63; 95% CI 1.23–5.64), age (HR 1.05; 95% CI 1.02–1.09), days since diagnosis until TCZ administration (HR 1.05, 95% CI 1.00–1.09), and platelets (HR 0.27; 95% CI: 0.11, 0.69). In the post-TCZ analysis and compared to discharged patients, deceased patients had more lactate dehydrogenase (*p* = 0.013), troponin I (*p* = 0.013), C-reactive protein (*p* = 0.013), neutrophils (*p* = 0.024), and fewer platelets (*p* = 0.013) and lymphocytes (*p* = 0.013) as well as a lower average PaO_2_/FiO_2_ ratio. In conclusion, in COVID-19 diagnosed patients receiving TCZ, early treatment decreased the risk of death, while age, some comorbidities and baseline lower platelet counts increased that risk. After TCZ administration, lower platelet levels were again associated with mortality, together with other laboratory parameters.

## Introduction

More than one year after the identification in December 2019 of a cluster of atypical pneumonia cases in Wuhan (China) caused by a new type of Coronavirus (SARS-CoV-2), the so called Coronavirus disease 2019 (COVID-19) pandemic is not under control despite the efforts of massive vaccination protocols. Therefore, the clinical management of non-immunized patients is still a big priority for the research community (Thoguluva Chandrasekar et al., 2021).

Male sex (Huang et al., 2020a), older age (Imam et al., 2020; Wu et al., 2020) and comorbidities (Imam et al., 2020) such as hypertension (Pranata et al., 2020), cardiovascular diseases (Aggarwal et al., 2020) and diabetes mellitus (Huang et al., 2020b) are risk factors for hospitalization and/or mortality in COVID-19 patients. Moreover, several analytical markers have been associated with severe COVID-19 disease and/or poor prognosis: elevated C-reactive protein (CRP), ferritin, procalcitonin, D-dimer, interleukin (IL)-6 and white blood cell levels as well as decreased albumin, lymphocyte and platelet levels (Zhang L. et al., 2020; Huang et al., 2020c; Henry et al., 2020; Yang et al., 2020). Of note, the alterations in D-dimer and platelet levels (together with other markers) may reflect hemostatic abnormalities similar to those occurring in the disseminated intravascular coagulopathy associated with sepsis (Huang et al., 2020c; Lippi et al., 2020; Tang et al., 2020).

In the severe stage of COVID-19 (Siddiqi and Mehra, 2020), shock, and respiratory and systemic organ failure may manifest secondary to a surge of proinflammatory cytokines (cytokine storm) which include IL-6, IL-1β, IL-2, granulocyte colony stimulating factor, macrophage inflammatory protein 1-α and tumor necrosis factor (Huang et al., 2020a; Siddiqi and Mehra, 2020; Wu et al., 2020). These cytokines increase vascular permeability facilitating the entrance of a large amount of fluid into the alveoli, thus causing dyspnea and respiratory failure (Zhang C. et al., 2020). IL-6 seems to have a prominent role in this stage. On the one hand, IL-6 binds to membrane IL-6 receptor (IL-6R) and induces the production of acute-phase proteins such as CRP and fibrinogen, biomarkers associated with poor COVID-19 outcomes (Siddiqi and Mehra, 2020). On the other hand, IL-6 binds to soluble IL-6R forming hyper-IL-6 which can activate all kind of cells, presenting a central role in the cytokine storm (Chastain et al., 2020). Thus, the use of IL-6R antagonists has been suggested as a potential therapy for severe COVID-19-related pneumonia cases.

Tocilizumab (TCZ) is an IL-6R antagonist that can effectively block the IL-6 signal transduction pathway. In observational studies, administration of TCZ to patients with pneumonia due to SARS-CoV-2 has been associated with higher survival rates (Gokhale et al., 2020; Rodríguez-Baño et al., 2020), and/or significant clinical improvement, including laboratory parameters like CRP (Madenidou and Bukhari, 2020; Toniati et al., 2020; Xu et al., 2020). Moreover, while some clinical trials did report no association of TCZ with clinical improvement (Roche Group Media Relations., 2020; Stone et al., 2020; Hermine et al., 2021; Salvarani et al., 2021; Veiga et al., 2021), those with a higher sample size did report a better COVID-19 outcome after TCZ treatment compared to the control group or null hypothesis (Perrone et al., 2020; Abani et al., 2021; Salama et al., 2021). Among the latter, it may be highlighted the RECOVERY trial, with 4,116 hospitalized COVID-19 patients, which showed that TCZ improved survival and other clinical outcomes regardless of the level of respiratory support (Abani et al., 2021). Thus, despite some initial controversy, the latest results seem to point TCZ as an efficacious treatment in severe COVID-19. Therefore, it is crucial to study the factors associated with better/worse outcomes as well as early markers of prognosis in COVID-19 patients under TCZ treatment and other therapies.

The aims of this study were to analyze the baseline predictors of hazard of death as well as the mean differences between discharged and deceased patients in several laboratory parameters measured in four consecutive tests before and after TCZ administration in a cohort of hospitalized patients with severe pneumonia or respiratory failure due to SARS-CoV-2 infection in Granada, Spain.

## Materials and Methods

### Study Design and Participants

This was a retrospective observational evaluation of all patients diagnosed with COVID-19 who received TCZ and were 18 years of age or older, admitted at Hospital Universitario Virgen de las Nieves (HUVN) in the city of Granada (southern Spain) between 13 March and November 5, 2020, coinciding with the peak of the second COVID-19 wave. COVID-19 diagnosis at admission included a positive polimerase chain reaction (PCR) test or a radiological and analytical suspicion. Patients were followed-up until hospital discharge or death.

The HUVN criteria to administer TCZ changed as the epidemic progressed and more knowledge was acquired. In March, TCZ was prescribed to patients with a severe hyperinflammatory syndrome, defined by severe bilateral pneumonia with criteria for acute respiratory distress syndrome (ARDS), or by the presence of two of the following criteria, fever >38.4°C, respiratory rate >24/min and Pa0_2_/Fi0_2_ <300 mmHg, and at least one analytical criterion of the following IL-6 >40 ng/L, D-dimer >1 mg/L and ferritin>300 μg/L. As of april, the criteria were two possible: 1) severe pneumonia with CRP >100 mg/L plus ARDS or Pa0_2_/Fi0_2_ <200 mmHg; 2) pneumonia in radiological progression, with progressive respiratory failure and/or progressive increase in CRP, D-dimer or ferritin, or progressive decrease in lymphocytes or presence of elevated IL-6. As of April 04, 2020, a single dose of TCZ of 600 mg in patients of ≥75 Kg and 400 mg in patients of <75 Kg was indicated. Previous protocols allowed administration of up to three doses in 72 h; thus, some patients of our cohort received more than one dose. Patient consent was obtained for the off-label use of TCZ.

All admitted patients received prophylactic doses of enoxaparin or bemiparin, adjusting for weight and renal function. In case of renal insufficiency, half of the weight-adjusted dose was used. This might be further adjusted according to anti-Xa levels. Anticoagulation with low molecular-weight heparin was started at intermediate doses (1 mg/Kg/day) if the patient had a high risk of thrombosis. Finally, if the patient had clinical suspicion of pulmonary embolism, low molecular-weight heparin was started or increased at therapeutic doses.

All data were fully anonymized before the analyses. The research was carried out according to The Code of Ethics of the World Medical Association (Declaration of Helsinki). The study was approved by the Research Ethics Committee of Granada, with waiver of informed consent due to the retrospective design and emergency of the research question.

### Variable Measurement and Definitions

The primary end point was time to death, defined as the time from administration of the first dose of TCZ until death. Censured data included hospital discharges. All the data used in this study were collected from electronic medical records for each patient.

#### General Information on Patient’s Admission

Sex; age (analyzed as continuous variable); COVID-19 wave (first or second); presence of comorbidities (hypertension, diabetes mellitus, dyslipidemia, previous pulmonary diseases such as asthma or chronic obstructive pulmonary disease, cardiovascular disease, immunosuppression like oncohematological tumor with active chemotherapy or immunosuppressive therapy); clinical findings on admission analyzed as dichotomic (yes/no) variables (fever, cough, fatigue/asthenia, dyspnea, headache, diarrhea, acute respiratory distress syndrome, acute cardiac injury, thrombosis, and acute renal injury); and smoking status (never smoker, current smoker, or ex-smoker).

#### Physical Examination and Laboratory Tests During Hospitalization

Information was collected before administration of TCZ (same or previous day) and 3, 6 and 9 days after TCZ administration. When a patient did not have a laboratory test in those specific dates, data from ± 1 day were used instead. The variables analyzed were: temperature (continuous variable), PaO_2_/FiO_2_ ratio, leukocyte count, lymphocyte count, neutrophil count, platelet count, total serum proteins, albumin, alanine transaminase, aspartate transaminase, γ-glutamyl transferase, lactate dehydrogenase (LDH), ferritin, CRP, procalcitonin, troponin I, D-dimer and fibrinogen. In addition, pre-TCZ X-ray findings were translated into a radiological scale for evaluation of patient admission (ERVI) (Catalá-Forteza, 2020).

#### Additional Pharmacological Treatment

Patients received the pharmacological standard treatment at the time of hospital admission, which changed throughout the pandemic: hydroxychloroquine, lopinavir/ritonavir, azithromycin and/or systemic methylprednisolone. Some patients received an additional pulse of methylprednisolone. Additionally, few patients received colchicine or cyclosporine or a dose of anakinra.

#### Other Variables

Place of hospitalization while administration of TCZ (general ward vs. ICU); time since symptoms onset until TCZ first dose administration; time since COVID-19 diagnosis until TCZ first dose administration; confirmed (by PCR) or suspicious diagnosis of COVID-19 with a negative PCR test on admission; and presence (yes/no) of a positive blood culture for secondary infection after TCZ administration.

### Statistical Analysis

A description of the baseline characteristics of the study participants was performed, reporting separately the patients who survived and those who died.

The variables alanine transaminase, aspartate transaminase, γ-glutamyl transferase, LDH, troponin I, CRP, procalcitonin, albumin, ferritin, leukocyte count, lymphocyte count, neutrophil count, platelet count, IL-6, D-dimer and PaO_2_/FiO_2_ ratio were log-transformed before regression analyses in order to reduce the skewness and the influence of extreme values.

Cox proportional hazard regression models were estimated in order to quantify the magnitude of associations between instantaneous death rate (measured as hazard ratio-HR-) and patients’ baseline characteristics. Because of the low ratio participant: independent variables, we used a three-step modeling process (Rivera-Izquierdo et al., 2020). First, univariate models were estimated for each predictive variable. Second, we defined subgroups of baseline variables (demographic, smoking status, COVID-19 wave, comorbidities and physical examination, pharmacological treatment, symptoms on hospital admission and laboratory values before TCZ administration). Then, we used a stepwise process to build multivariate models for each group, including all the variables with a *p*-value <0.2 in the univariate analyses except variables with >10% of missing values that could compromise the statistical power. Third, the variables retained in each group model were incorporated in a new stepwise regression to build a final model. In all stepwise regressions performed, those variables with *p*-value <0.05 were sequentially retained in the model and those with *p*-value ≥0.10 were excluded from it. We calculated for each HR the 95% confidence intervals (CI).

The mean change in the 18 parameters along the four laboratory tests (baseline, and days 3, 6 and 9) was analyzed, considering the death during follow-up (yes/no) as the independent variable. For this analysis, weighted generalized estimation equations were calculated using the *xtrccipw* command in the statistics software Stata, which allowed the truncation of deaths along the follow-up (Daza et al., 2017). An adjustment of the *p*-value because of multiple comparisons was performed by means of the Benjamini-Hochberg method.

A *p*-value ≤ 0.05 was set for the level of statistical significance. Statistical analyses were performed using the statistics software Stata v.15 (Stata Corp, 2017) and graphs were built using Graph Pad v.8.4.3.

## Results

A total of 120 patients diagnosed with COVID-19 received TCZ at HUVN during the recruitment period. By the end of the follow-up period, 86 (72%) had been discharged and 34 (28%) had deceased with a mean time to death (from TCZ administration) of 15.9 days and a standard deviation (SD) of 16.6 days. Baseline demographic, clinical and pharmacological characteristics, as well as laboratory parameters, are shown in Table 1 for the whole cohort as well as for the groups of patients that died and survived respectively. On the one hand, median LDH, albumin, CRP, IL-6 and D-dimer levels were above normal values on both deceased and discharged patients, and median troponin I level only in the group of patients that died. Of those patients with high Troponin-I levels (above 20 pg/dl), only four had records of clinical cardiologic affectation: 2 with arrhythmia, 1 with ST segment depression and 1 with a hyperdynamic left ventricle.

**TABLE 1:** Baseline demographic, clinical, pharmacological and laboratory data of patients diagnosed with COVID-19 receiving tocilizumab.

	**Total (*N* = 120)**	**Deceased (*N* = 34)**	**Survivors (*N* = 86)**
Sociodemographic variables			
Age, mean (SD)	63.0 (13.8)	68.2 (14.3)	61.0 (13.1)
Sex (men), *n* (%)	86 (71.7)	28 (82.4)	58 (67.4)
COVID-19 wave (first), *n*(%)	59 (49)	15(44)	44 (51)
Smoking status, *n* (%)*			
Non-smoker	50 (52.1)	9 (40.9)	41 (55.4)
Smoker	6 (6.2)	3 (13.6)	3 (4.1)
Ex-smoker	40 (41.7)	10 (45.5)	30 (40.5)
Comorbidities and physical examination, *n* (%)			
Hypertension	65 (54.2)	22 (64.7)	43 (50.0)
Dyslipidemia	50 (41.7)	20 (58.8)	30 (34.9)
Cardiovascular disease	49 (40.8)	18 (52.9)	31 (36.1)
Diabetes mellitus	23 (19.2)	11 (32.4)	12 (13.9)
Previous pulmonar disease	25 (20.8)	10 (29.4)	15 (17.4)
Immunosuppression	13 (10.8)	8 (23.5)	5 (5.8)
Diagnosis by PCR			
Confirmed	98 (81.7)	26 (76.5)	72 (83.7)
Suspicion	22 (18.3)	8 (23.5)	14 (16.3)
Pharmacological treatment, *n* (%)			
Lopinavir/ritonavir	61 (50.8)	15 (44.1)	46 (53.5)
Hydroxychloroquine	59 (49.2)	15 (44.1)	44 (51.2)
Azytromicine	62 (51.7)	14 (41.2)	48 (55.8)
Methylprednisolone	107 (89.2)	30 (88.2)	77 (89.5)
Pulses of methylprednisolone	86 (72.9)	20 (60.6)	66 (77.6)
Cyclosporine	2 (1.7)	1 (2.9)	1 (1.2)
Colchicine	3 (2.5)	1 (2.9)	2 (2.3)
Anakinra	13 (10.8)	9 (10.5)	4 (11.8)
TCZ characteristics			
Days since symptoms until TCZ, mean (SD)^*^	10.9 (4.6)	9.5 (6.0)	11.4 (3.8)
Days since diagnosis until TCZ, mean (SD)	4.8 (7.6)	6.7 (13.5)	4.1 (3.0)
Second dose of TCZ, *n* (%)	28 (23.3)	9 (26.5)	19 (22.1)
Third dose of TCZ, *n* (%)	2 (1.7)	1 (2.9)	1 (1.2)
Hospitalization when TCZ administration			
General ward, *n* (%)	91 (75.8)	23 (67.6)	68 (79.1)
ICU, *n* (%)	29 (24.2)	11 (32.4)	18 (20.9)
Clinical findings on admission, *n* (%)			
Fever	99 (82.5)	28 (82.4)	71 (82.6)
Dry cough	90 (75.0)	25 (73.5)	65 (75.6)
Fatigue	67 (55.8)	17 (50.0)	50 (58.1)
Myalgia	45 (37.5)	6 (17.6)	39 (45.3)
Dyspnea	82 (68.3)	21 (61.8)	61 (70.9)
Headache	15 (12.5)	4 (11.8)	11 (12.8)
Diarrhea	14 (11.7)	2 (5.9)	12 (13.9)
ARDS	24 (20.0)	9 (26.5)	15 (17.4)
ACI	3 (2.5)	2 (5.9)	1 (1.2)
Thrombosis	4 (3.3)	1 (2.9)	3 (3.5)
Secondary infection	10 (8.3)	3 (8.8)	7 (8.1)
ARI	9 (7.5)	5 (14.7)	4 (4.6)
ERVI Scale*	5.5 (1.7)	5.6 (1.6)	5.4 (1.7)
Laboratory findings pre-TCZ, median (IQR) [N] except when indicated			
Total serum proteins (gr/dL), mean (SD) [N]	6.5 (0.8) [99]	6.3 (0.8) [28]	6.6 (0.8) [71]
Albumin (gr/dL)	3.4 (3–3.8) [91]	3.3 (3–3.5) [25]	3.4 (3–3.9) [66]
AST (U/L)	38 (26–54) [116]	37 (25–54) [33]	38 (26–54) [83]
ALT (U/L)	36 (23–66) [118]	28.5 (22–52) [34]	37.5 (25–71) [84]
GGT (U/L)	67.5 (47–110) [86]	54.5 (44–78) [26]	73 (51–129) [60]
LDH (U/L)	476 (392–573.5) [120]	490 (409–574) [34]	473 (391–566) [86]
Troponin I (pg/ml)	6.4 (2.7–17.6) [80]	18.8 (6–82.7) [26]	4.6 (2.4–9.5) [54]
C-reactive protein (mg/L)	119.9 (67.2–182.7) [118]	133.3 (80.3–182.7) [33]	107.1 (67.2–182.5) [85]
Procalcitonine (ng/ml)	0.2 (0.0–0.5) [80]	0.2 (0.1–0.5) [26]	0.2 (0.1–0.4) [54]
Ferritin (ng/ml)	1,415.7 (812.9–2,475) [114]	1748.9 (865.1–2,811.8) [32]	1,322.1 (794.6–2,202.6) [82]
Leukocyte count (/µL)	8,480 (6,390–11,330) [120]	8,255 (5,560–12,420) [34]	8,545 (6,760–11,200) [86]
Neutrophil count (/µL)	7,050 (4,900–9,930) [119]	6,760 (4,800–9,780) [33]	7,690 (5,020–9,930) [86]
Lymphocyte count (/µL)	690 (430–990) [119]	660 (380–970) [33]	695 (460–990) [86]
Platelet count (/µL)	228,500 (179,000–293,500) [120]	193,500 (140,000–242,000) [34]	245,500 (199,000–343,000) [86]
Interleukin-6 (pg/ml)	74.6 (39.6–133.3) [80]	78.4 (37.8–240.2) [23]	65.8 (40–111.7) [57]
D-Dimer (mg/L)	1.0 (0.6–2.3) [113]	1.1 (0.6–3.0) [32]	1.0 (0.6–2.3) [81]
Fibrinogen (mg/dl), mean (SD) [N]	725.9 (274.8) [85]	752.0 (305.5) [28]	726.4 (261.2) [57]
PaO_2_/FiO_2_ ratio	180 (163–252.5) [120]	171 (138–200) [34]	193.5 (172–263) [86]
Temperature (°C), mean (SD) [N]	36.8 (1.1) [60]	36.1 (0.9) [14]	36.9 (1.1) [46]

ACI, acute cardiac injury; ARDS, acute respiratory distress syndrome; ACI, acute cardiac injury; ARI, acute renal injury; AST, Aspartate aminotransferase; ALT, Alanine aminotransferase; ERVI, scale for assessment of hospital admission; GGT, γ-glutamyl transferase; IQR, interquartile range; LDH, lactate dehydrogenase; SD, Standard deviation.

* Variables with missing values: Smoking status *N* = 96, *N*_*deceased*_ = 22, *N*_*survivors*_ = 74; Days since symptoms until TCZ *N* = 118, *N*_*deceased*_ = 32, *N*_*survivors*_ = 86; ERVI Scale *N* = 116, *N*_*deceased*_ = 33, *N*_*survivors*_ = 83.

On the other hand, mean PaO_2_/FiO_2_ ratio as well as lymphocyte count were below the normal range in both groups. Mean platelet count was much lower in the patients that died compared to those that remained alive, but in both case values entered into the normal range. Central tendency and dispersion values together with sample size for the analytical parameters measured at day 3, 6 and 9 are provided in Supplementary Table S1.

The univariate analyses revealed a statistically significant positive association of age, diabetes mellitus, immunosuppression, troponin I levels, and days since diagnosis until TCZ administration with hazard of death (Table 2). Instead, myalgia on admission, temperature, platelet count and total serum proteins were significantly related with a lower hazard of death (Table 2).

**TABLE 2:** Association between baseline variables and time to death.

**Group variables**	**Variable**	**HR^c^**	**95%CI**	***p*-value**	**HR^d^**	**95%CI**	***p*-value**	**HR^e^**	**95%CI**	***p*-value**
Demographic variables	Male	1.68	0.70–4.08	0.248						
	**Age (years)** ^a^	**1.04**	**1.01**–**1.07**	**0.015**				**1.05**	**1.02**–**1.09**	**0.001**
COVID-19 wave	Second	1.26	0.64–2.50	0.503						
Smoking habit	Smokers	2.24	0.60–8.34	0.231						
	Ex-smokers	1.04	0.41–2.63	0.930						
Comorbidities and physical examination	Hypertension	1.60	0.79–3.24	0.196						
	Dyslipidemia	1.70	0.86–3.38	0.128						
	Cardiovascular disease	1.51	0.77–2.98	0.230						
	**Diabetes mellitus**	**2.26**	**1.09**–**4.68**	**0.029**	**2.25**	**1.08**–**4.69**	**0.030**	**2.63**	**1.23**–**5.64**	**0.013**
	Previous pulmonar disease	1.44	0.68–3.03	0.340						
	**Immunosuppression**	**4.85**	**2.15–10.95**	**<0.001**	**4.87**	**2.15–11.07**	**<0.001**	**3.15**	**1.17–8.51**	**0.024**
	Confirmed diagnosis by PCR	0.59	0.26–1.33	0.204						
Pharmacological treatment	Lopinavir/ritonavir	0.75	0.38–1.48	0.407						
	Azytromicine	0.85	0.43–1.69	0.640						
	Anakinra	0.99	0.35–2.83	0.993						
	Methylprednisolone	0.93	0.33–2.66	0.899						
	Ciclosporine	2.59	0.35–19.36	0.354						
	Pulses of methylprednisolone	0.85	0.42–1.72	0.644						
	Colchicine	1.27	0.17–9.35	0.814						
	Hydroxycloroquine	0.79	0.40–1.57	0.507						
	More than one dose of TCZ	1.03	0.46–2.29	0.941						
	Days since symptoms until TCZ^a^	0.93	0.86–1.02	0.117						
	**Days since diagnosis until TCZ** ^a^	**1.06**	**1.03–1.09**	**<0.001**	**1.07**	**1.03–1.10**	**<0.001**	**1.05**	**1.00–1.09**	**0.032**
Symptoms and signs pre-TCZ	Dry cough	1.00	0.47–2.16	0.991						
	Fatigue	0.75	0.38–1.48	0.411						
	**Myalgia**	**0.35**	**0.15–0.86**	**0.021**	**0.35**	**0.15–0.86**	**0.021**			
	Dyspnea	0.69	0.35–1.38	0.294						
	Headache	0.70	0.25–2.00	0.511						
	Diarrhea	0.68	0.16–2.87	0.602						
	Acute Respiratory distress syndrome	0.68	0.30–1.53	0.353						
	Acute cardiac injury	3.22	0.76–13.64	0.113						
	Thrombosis	0.88	0.12–6.43	0.897						
	Acute renal injury	1.95	0.75–5.08	0.172						
	Scale ERVI^a^	1.02	0.83–1.25	0.858						
	Hospitalization when TCZ administration	0.64	0.30–1.35	0.243						
	Fever	0.62	0.25–1.53	0.301						
Laboratory findings pre-TCZ	**Total serum proteins (gr/dL)** ^b^	**0.53**	**0.31–0.92**	**0.025**						
	Aspartate transaminase (U/L)^b^	0.78	0.39–1.56	0.481						
	Alanine transaminase (U/L)^b^	0.55	0.30–1.00	0.052						
	γ-Glutamyl transferase (U/L)^b^	0.67	0.38–1.18	0.161						
	Procalcitonin (ng/ml)^b^	0.97	0.74–1.26	0.804						
	Albumin (gr/dL)^b^	0.40	0.05–3.50	0.409						
	Interleukin-6 (pg/ml)^b^	1.08	0.79–1.48	0.611						
	Lactate deshydrogenase (U/L)^b^	1.87	0.55–6.29	0.312						
	C-reactive protein (mg/L)^b^	0.93	0.66–1.31	0.669						
	Ferritin (ng/ml)^b^	1.24	0.82–1.88	0.302						
	Leukocyte count (/μL)^b^	0.69	0.31–1.54	0.368						
	Neutrophil count (/μL)^b^	0.69	0.32–1.48	0.343						
	Lymphocyte count (/μL)^b^	0.88	0.47–1.65	0.695						
	**Platelet count (/μL)** ^b^	**0.20**	**0.10–0.40**	**<0.001**	**0.20**	**0.10–0.41**	**<0.001**	**0.27**	**0.11–0.69**	**0.006**
	Fibrinogen (mg/dl)^b^	1.00	1.00–1.00	0.440						
	**Troponin I (pg/ml)** ^b^	**1.35**	**1.08–1.69**	**0.007**						
	D-dimer (mg/L)^b^	1.15	0.86–1.52	0.342						
	PaO_2_/FiO_2_ (mmHg)^b^	0.79	0.30–2.25	0.663						
	**Temperature°C** ^a^	**0.39**	**0.21–0.76**	0.005						

PCR: polymerase Chain Reaction; Scale ERVI: X-ray scale for assessment of hospital admission; TCZ: Tocilizumab; HR: hazard ratio; CI: confidence interval. For dichotomous variables, the reference category was “no” except hospitalization when TCZ administration that it was “general ward” (comparison ICU) and confirmed diagnosis-PCR that it was “suspicious diagnosis with negative PCR” (comparison confirmed positive PCR result). For smoking habit the reference was “never smoker”. Highlighted in bold if p < 0.05.

^a^Hazard ratios are expressed per unit increase in the variable.

^b^Log-transformed quantitative variables.

^c^Obtained with Cox’s univariate proportional hazard regression models.

^d^Obtained with stepwise regression model within groups of variables, including variables with *p*-value <0.2 in univariate analysis, except total serum proteins (N = 99), troponin I (*N* = 80) and temperature (*N* = 60) which were excluded from multivariate models because of presenting >10% of missing values.

^e^Obtained with stepwise regression including variables retained in models by group.

**FIGURE 1:**
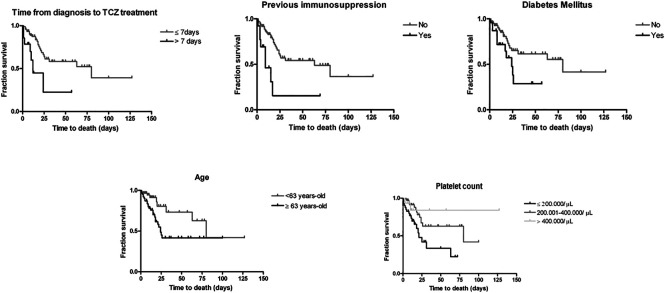
Survival curves of the variables associated with time to death. Censored (discharged by the end of follow-up) subjects are indicated on the curve as tick marks.

In Cox stepwise regression models within each group of factors, diabetes mellitus, immunosuppression, days since diagnosis until TCZ administration, myalgia and platelets were the variables retained. In the final stepwise regression model, the variables associated with a higher hazard of death were age (for each year of increase in age, HR 1.05; 95% CI 1.02–1.09), diabetes mellitus (HR 2.63; 95% CI 1.23–5.64), days since diagnosis until TCZ administration (for each more day, HR 1.05, 95% CI 1.00–1.09) and immunosuppression (HR 3.15; 95% CI 1.17–8.51). The immunosuppressed group included five patients with haematological neoplasms and five with other type of neoplasms under active treatment with chemotherapy, two transplant recipients with immunosuppressive treatment and one patient with an autoimmune disease and under treatment with biological therapy and methotrexate. Furthermore, for every logarithmic unit increase in platelet count there was a 73% decrease in the instantaneous death rate (HR 0.27; 95% CI 0.11–0.69). Survival curves illustrating the variables associated with time to death in the final regression model are represented in Figure 1. For continuous variables, population was divided in groups. In days from diagnosis until TCZ treatment, two groups are showed: ≤7 days and >7 days. For age, two groups were built with individuals below/above the median (63 years). And for platelets, three groups: ≤ 200,000, 200,000–400000, ≥400,000/μL.

With regards to the changes in biochemical and hemogram parameters after TCZ administration and comparing with participants who remained alive during the follow-up, participants who died had a significant positive mean difference (higher mean values along the four measurements) in LDH, troponin I, CRP, procalcitonin, neutrophils, D-dimer, IL-6 and leukocytes (Table 3). After adjustment for multiple comparisons, the parameters that remained significant were LDH, troponin I, CRP and neutrophils, while a borderline non-significant association was retained for IL-6. The mean difference was negative (lower values in the deceased patients) for PaO_2_/FiO_2_ ratio, and lymphocyte and platelets counts. The statistically significance was kept for the three parameters after adjustment (Table 3).

**TABLE 3:** Mean differences in the biochemical markers and hemogram parameters between individual with COVID-19 according to the vital status.

Variable		Mean difference (95% CI)^a^	*p*-value		*q*-value
Lactate Dehydrogenase (U/L)^b^		0.26 (0.13, 0.39)	**<0.001**		**0.013**
Troponin I (pg/ml)^b^		1.58 (0.78, 2.37)	**<0.001**		**0.013**
C Reactive protein (mg/L)^b^		0.76 (0.31, 1.22)	**0.001**		**0.013**
Procalcitonin (ng/ml)^b^		0.77 (0.18, 1.36)	**0.010**		0.100
Neutrophils (/μl)^b^		0.27 (0.10, 0.45)	**0.002**		**0.024**
D-dimer (mg/L)^b^		0.59 (0.10, 1.09)	**0.019**		0.152
Interleukin-6 (pg/ml)^b^		1.41 (0.37, 2.44)	**0.008**		0.088
Albumin (gr/dL)^b^		−0.05 (−0.09, −0.00)	0.058		0.406
Platelet count (/μl)^b^		−0.30 (−0.48, −0.12)	**0.001**		**0.013**
Aspartate transaminase (U/L)^b^		0.12 (−0.07, 0.32)	0.213		0.679
Alanine transaminase (U/L)^b^		−0.13 (−0.42, 0.16)	0.386		0. 679
γ-Glutamyl transferase (U/L)^b^		−0.12 (−0.52, 0.28)	0.556		0. 679
Ferritin (ng/ml)^b^		0.37 (−0.03, 0.77)	0.070		0.420
Leukocyte count (/μL)^b^		0.23 (0.05, 0.41)	**0.013**		0.117
Lymphocyte count (/μL)^b^		−0.38 (−0.58, −0.18)	**<0.001**		**0.013**
Total serum proteins (gr/dL)		−0.19 (−0.44, 0.05)	0.124		0.577
PaO_2_/FiO_2_ ratio^b^		−0.27 (−0.37, −0.17)	**<0.001**		**0.013**
Fibrinogen (mg/dl)		17.56 (−65.48,100.60)	0.679		0.679

a Mean difference between survivors and deceased along four laboratory measurements (days 0, 3, 6 and 9 after TCZ administration) using generalized estimating equation;

b Parameters analyzed in logarithmic units; CI: confidence interval; *q*-value: *p*-value adjusted by multiple comparisons with Benjamini-Hochber method. In bold if *p*-value < 0.05.

**FIGURE 2:**
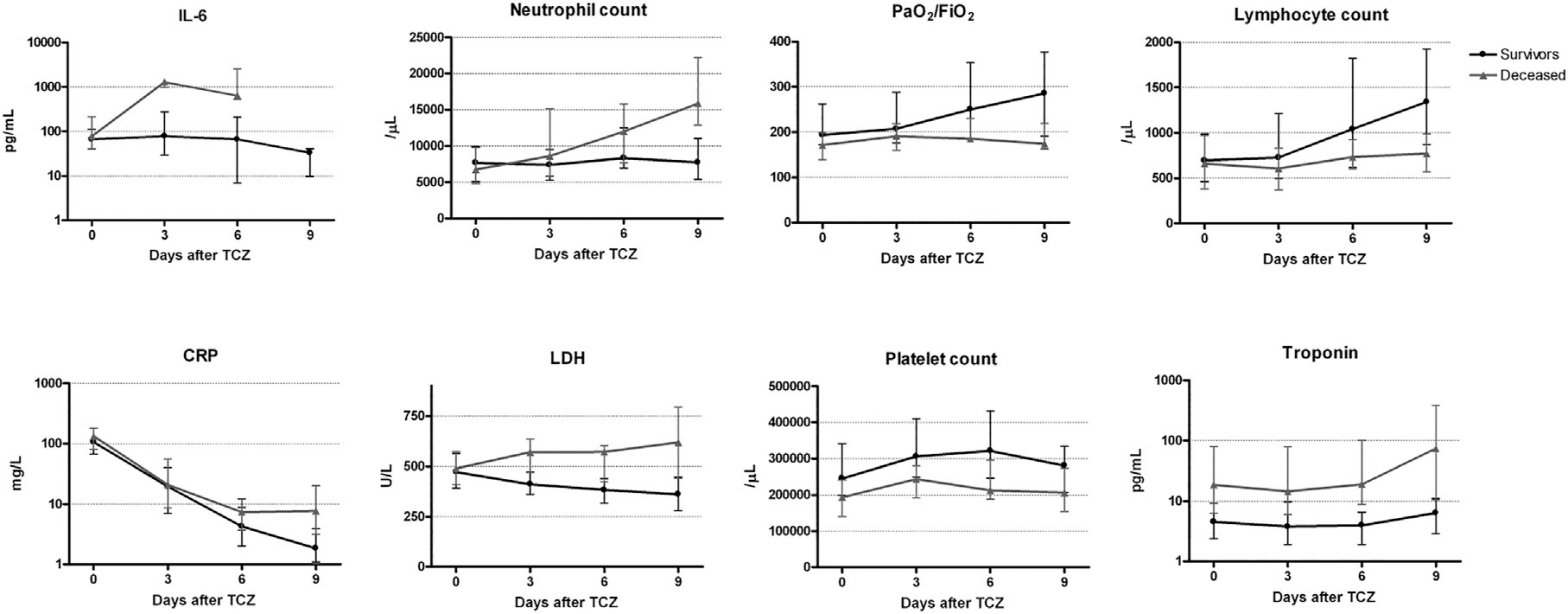
Changes in laboratory parameters after tocilizumab administration and their comparison between survivors and deceased patient. LDH: Lactate dehydrogenase; CRP: C-reactiveprotein; IL-6: interleukin-6. Values are represented as median (circle) and interquartile range (25th and 75th percentiles error bars).

Kinetics varied depending on the parameter (Figure 2). On the one hand, IL-6, PaO_2_/FiO_2_ ratio, lymphocyte and neutrophil counts, CRP and LDH showed differences between survivors and deceased patients that were amplified over time. Thus, IL-6 and neutrophil counts increased only in the deceased population, reaching pathological median levels in the case of neutrophils by day 3. Lymphocyte count and PaO_2_/FiO_2_ ratio values improved only in the survivors, entering the physiological range by day 6 in the case of the lymphocytes. CRP decreased in both groups but at a higher rate in the survivors, such as that the median level reached the physiological levels (<5 mg/L) by day 6. For LDH an increase was observed in the deceased and a decrease in the survivor group. On the other hand, troponin I and platelet counts presented baseline differences between both groups of comparisons that were kept over the four measurements. Of note, platelet counts increased at day 3 and 6 and then decreased both in deceased and discharged patients; however, levels were always lower in the patients that died. Contrary, troponin I levels were higher in deceased patients at all four time points.

Finally, as one adverse effect of TCZ treatment is the risk of bacterial infections, we hypothesized that immunosuppressed individuals receiving TCZ may be at higher risk of death precisely because of secondary co-infections. In our cohort, 38 patients (31.7%) presented a secondary systemic infection after TCZ administration, of which 18 (47%) died. Post-TCZ coinfections were associated with mortality (χ^2^ test, *p*-value = 0.002) and were more common in the ICU than in the general ward (χ^2^ test, *p*-value<0.001). However, the percentage of post-TCZ coinfections was similar between immunosuppressed and non-immunosuppressed individuals (Fisher’s test, *p*-value = 0.481). Therefore, we also searched actively for any record of *Aspergillus* spp. growth in broncho-alveolar aspirates in patients hospitalized at the ICU. None of them was positive for this pathogen.

## Discussion

There were more men than women in our cohort, and the percentage skewed even more in the deceased group, a common observation in COVID-19. However, in agreement with previous TCZ-cohorts of COVID-19, sex was not associated with the risk of death (Moreno-Pérez et al., 2020; Morrison et al., 2020; Desai et al., 2021). On the contrary, risk death did increase with age, while literature shows contradictory results (Moreno-Pérez et al., 2020; Morrison et al., 2020; Desai et al., 2021).

In our study, the comorbidities associated with a higher hazard of death were immunosuppression and diabetes mellitus. Diabetes mellitus has been largely associated to poorer outcomes in COVID-19 patients [reviewed in (Huang et al., 2020b)], while contradictory results have been observed in cohorts under TCZ treatment (Moreno-Pérez et al., 2020; Morrison et al., 2020; Desai et al., 2021). Obesity has been related to a higher risk of death in COVID-19 patients (Demeulemeester et al., 2021) and may act as a cofounder or modifier variable for this finding. Unfortunately, as we collected the information retrospectively from clinical records where obesity was not codified, we could not analyze its effect in our death risk estimation. With regards to immunosuppression, it was not associated with mortality in a large COVID-19 cohort (*N* = 1,305) in the United States (Imam et al., 2020). However, the effect of this condition in COVID-19 risk/mortality may depend on the type of immunosuppression. Thus, while cancer and solid organ transplant patients seem to present higher rates of mortality (Belsky et al., 2021) and autoinmune diseases’s patients have a higher risk of COVID-19 infection (Akiyama et al., 2021), people living with Human Immunodeficiency Virus were not found to be at higher risk of poorer COVID-19 outcomes (Lee et al., 2021). In TCZ-COVID-19 cohorts, the effect of immunosuppression was not estimated (Moreno-Pérez et al., 2020; Morrison et al., 2020) or the variable was included in the group of comorbidities, precluding a specific analysis (Lohse et al., 2020; Galván-Román et al., 2021). Nevertheless, a publication recently reported a higher risk of death among cancer patients receiving TCZ due to COVID-19, but not among patients with previous rheumatology/infectious diseases (Desai et al., 2021). Due to our study design, we cannot conclude whether the TCZ-induced immunosuppression acted as an added risk factor for death in previously immunocompromised patients. However, it seems unlikely as coinfections were not more frequent in immunosuppressed individuals in our cohort. Further studies are necessary to confirm our finding and to provide knowledge about a potential underlying mechanism.

Time elapsed from COVID-19 diagnosis to TCZ was positively associated with the risk of death, as previously reported (Morrison et al., 2020). Of note, when we stratified our cohort into patients with a PaO_2_/FiO_2_ ratio lower and higher or equal to 200, the effect of time to treatment on mortality was only observed in those with PaO_2_/FiO_2_ ratio <200 (data not shown), so timing seems specially important in moderate/severe disease. In a similar direction, Galván-Román et al. reported that early TCZ administration improved the PaO_2_/FiO_2_ ratio (Galván-Román et al., 2021). The effect in our study was clearly observed when we classified patients in early (within a week of diagnosis) and late (after 7 days) TCZ treatment. This time window is in consonance with the studies referenced above, that established it in 11–12 days since symptoms onset (Morrison et al., 2020; Galván-Román et al., 2021), which usually occurs days before diagnosis. In our study, time from symptoms to TCZ did not show significant differences, probably as the first day of symptoms is often not recorded properly as they are usually mild and vague. Nevertheless, our results emphasize the importance of the appropriate timing to administer TCZ, which may explain the contradictory results about its efficacy reported by observational and experimental studies from the literature.

Our cohort included patients hospitalized in the general ward (less severe disease) and in the ICU at the time of TCZ administration. Surprisingly, mortality risk was similar between both groups, suggesting that initial clinical differences were not related to a poorer prognosis. Nevertheless, post-TCZ coinfections were much more likely to occur at ICU as expected (Quartuccio et al., 2020), and they tended to be associated with mortality in contrast to a previous study with a TCZ-cohort (Morrison et al., 2020). In COVID-19 TCZ-cohorts, percentages of patients who developed secondary infections have ranged from 10 to 40% (Alattar et al., 2020; Moreno-Pérez et al., 2020; Pérez-Sáez et al., 2020; Quartuccio et al., 2020), what covers the 32% presented here.

Baseline levels of ferritin, CRP and procalcitonin have been related to mortality or poor outcomes in hospitalized COVID-19 patients (Bonetti et al., 2020; Huang et al., 2020c) but not in our final regression model, in agreement with other COVID-19 –TCZ cohorts (Conrozier et al., 2020; Knorr et al., 2020). However, after TCZ administration, the longitudinal laboratory test analysis showed that CRP decreased differentially in survivors and deceased patients, indicating that TCZ was more effective controlling inflammation in those patients that remained alive at the end of the study. This is in consonance with other studies (Alattar et al., 2020; Antwi-Amoabeng et al., 2020; Conrozier et al., 2020; Knorr et al., 2020; Morrison et al., 2020; Pérez-Sáez et al., 2020) and suggests that CRP may be used as a prognostic biomarker after TCZ administration in COVID-19 severe patients.

IL-6 has been recognized as another key inflammatory marker in COVID-19 and a meta-analysis has shown elevated levels in patients with complicated COVID-19 (Coomes and Haghbayan, 2020). Here, it was not found an association between baseline IL-6 levels and death, probably because these levels were already elevated in all patients, suggesting an adequate used of its antagonist TCZ. Interestingly, we report that IL-6 levels increased massively in the following days after TCZ administration only in the patients that subsequently deceased, but not in those that remained alive where we observed just a small spike at day 3. Other authors have found a similar trend for IL-6 concentration differences between deceased and discharged patients but with no statistical analysis for between-group comparison (Luo et al., 2020; Madenidou and Bukhari, 2020; Toniati et al., 2020). However, it is unclear whether IL-6 represents a marker and/or mediator of COVID-19 severe progression (Chastain et al., 2020). This finding was accompanied by an increased on neutrophils few days after the IL-6 peak, as it is well recognized that IL-6 stimulates neutrophil production in the bone marrow (Abbas et al., 2018). Indeed, while there were no baseline differences in neutrophil numbers between comparison groups and levels were within physiological values, their median value reached pathological values after TCZ only in the deceased group. The neutrophil count is probably a more easily measurable, available and cost-effective parameter than IL-6 and therefore may be used as a prognostic IL-6 proxy factor.

LDH is a well-known marker of tissue damage, and in our study it was another of the parameters showing differences between discharged and deceased patients early after TCZ administration, in consonance with a previous study (Morrison et al., 2020). Of note, baseline levels were higher than normal and very similar in both groups, and no association with time to death was observed. However, 3 days after TCZ administration, an increase in LDH levels was observed in the patients that subsequently died, suggesting further tissue damage could be occurring in these patients. The PaO_2_/FiO_2_ ratio is a marker of severity of acute respiratory distress syndrome, a common and severe complication of COVID-19 (Badraoui et al., 2020), considered moderate if the values range between 100 and 200, and severe if <100. There were basically no differences in the baseline values between patients that died and survived on our cohort and no association with mortality at this stage. However, after TCZ, a progressive increase in the ratio values was observed only in the survivor group, suggesting a pulmonary improvement in agreement with the better clinical outcome. Similarly, median lymphocyte levels were below the normal range in both groups before TCZ treatment, but after the treatment, only the survivors increased their counts.

Troponin I showed an association with risk of death in the univariate analysis. However, because of having many missing values, this variable was excluded from the multivariate analysis, precluding the opportunity to study its effect in the global regression model. Nevertheless, differences between deceased and survivors were also observed along the four measurements after TCZ treatment. Plasma troponin I is a marker of cardiac muscle damage and/or myocarditis and its levels have been related to poor COVID-19 outcomes [reviewed in (Alzahrani and Al-Rabia, 2021)]. Its role in our cohort is probably independent of the effect of TCZ.

The variable most clearly (inversely) associated with mortality was platelet count, as baseline as well as the mean longitudinal change post-TCZ was associated with mortality. Corticosteroid treatment was not associated to differences in baseline platelet levels (data not shown). Unfortunately, we did not collect information about concomitant treatment with other potential drugs altering platelet levels such as anticoagulants because at the time of the study design evidence for the role of coagulation in COVID-19 was not so strong. We cannot rule out that this was a bias in our study, as the patients at risk of thrombosis were more likely to die but also more likely to receive anticoagulant treatment that may decrease platelet count. Nevertheless, in agreement with our results, thrombocytopenia as well as lower platelet count has been repeatedly related to poor COVID-19 outcomes, in general hospitalized cohorts [reviewed in (Lippi et al., 2020)] as well as a TCZ-cohort (Conrozier et al., 2020). Thus, an increase in platelet counts after any clinical or pharmacological intervention might be understood as a positive sign. However, here we report that regardless of the health outcome (live or death), an early increase in the platelet count occurred 3 and 6 days after TCZ administration followed by a decrease, in consonance with a longitudinal analysis of a similar cohort (Conrozier et al., 2020). This temporarily increase may mislead practitioners about the disease outcome and suggest that total platelet count rather than progression should be taken into account when interpreting this parameter in relation to COVID-19 progression. Other hemostasis alterations reflecting intravascular or consumption coagulopathies are common in COVID-19 (Bonetti et al., 2020; Huang et al., 2020c; Tang et al., 2020). In contrast with some of these studies, we did not find an association between baseline D-dimer or fibrinogen concentration and mortality. And while significant mean post-TCZ differences were observed between deceased and discharged patients for D-dimer, the significance was lost when we adjusted for multiple comparisons.

Our study has some limitations: 1) A possible lack of statistical power due to the relatively low sample size and the presence of missing values for some laboratory parameters. Nevertheless, to allow the analysis of all the variables recorded despite the small sample size, we did a three-stage modeling process grouping predictors and reducing the number of variables included in a regression model at a time. 2) The absence of a control group as patients with no TCZ treatment would not be clinically comparable (less severe disease). 3) In this retrospective study, the information source were the clinical records and therefore the effect of possible relevant variables, e.g. the obesity, could not be evaluated due to not having been routinely registered.

This study has also important strengths. Although few similar articles in COVID-19 patients under TCZ treatment have been published (many of them referenced along the manuscript), our statistical approach was different as it allowed the analysis of multiple variables resulting in a model with those that contributed most to mortality. Furthermore, while most studies focused on the baseline predictors of mortality, we also analyzed the laboratory parameter evolution early after TCZ administration and how this evolution differed between discharged and deceased patients. Finally, our larger follow-up period allowed us to observe the final outcome (discharge or death) of the whole cohort.

As conclusions, our results show that in a cohort of COVID-19 diagnosed patients under TCZ treatment, early treatment decreased the risk of death, while age, immunosupression, diabetes mellitus and baseline lower platelet counts increased that risk. Lower platelet levels were also associated with mortality after TCZ administration, while increased troponin I values were observed in the deceased patients. Moreover, IL-6, neutrophil and lymphocyte count, PaO_2_/FiO_2_ ratio, LDH and CRP evolved differently in deceased and discharged patients after TCZ treatment, and may be used as prognostic factors in these patients.

## Data Availability

The original contributions presented in the study are included in the article/Supplementary Material, further inquiries can be directed to the corresponding authors.
