# Supplementary material for: Tocilizumab in COVID-19: Factors Associated With Mortality Before and After Treatment
Source: Front Pharmacol. 2021 Jul 1;12:620187. doi: 10.3389/fphar.2021.620187 (PMC8281134; doi:10.3389/fphar.2021.620187)
Supplement: Supplementary file 1 [file Table1.pdf]

Supplementary Material

Supplementary Table 1. Description of analytical parameters measured at day 3, 6 and 9 of patients diagnosed of COVID-19 and receiving tocilizumab (TCZ).

**Laboratory/clinical findings** **3 days post-TCZ,** median (IQR) [N] except when indicated	**Total**	**Deceased**	**Survivors**
Total serum proteins (gr/dL), mean (SD)	6 (0.7) [81]	5.7 (0.7) [19]	6 (0.7) [62]
Albumin (gr/dL)	3.2 (2.9-3.6) [72]	3 (2.7-3.2) [18]	3.3 (3-3.6) [54]
AST (U/L)	36 (24-47) [99]	43.5 (33-68) [24]	33 (23-46) [75]
ALT (U/L)	53 (30-95) [101]	49.5 (35.5-73) [24]	54 (29-96) [77]
GGT (U/L)	117 (69-209) [73]	104 (72-181) [17]	117.5 (65-209.5) [56]
LDH (U/L)	435 (367-544) [99]	570.5 (464-643.5) [24]	411 (361-473) [75]
Troponin I (pg/mL)	4.9 (2-13.6) [59]	14.6 (5.2-81.9) [14]	3.8 (1.9-9.7) [45]
CRP (mg/L)	19.8 (7.6-41.5) [102]	20.9 (8.7-55.9) [25]	19.6 (7-40.3) [77]
Procalcitonine (ng/mL)	0.1 (0.0-0.2) [72]	0.1 (0.1-0.2) [18]	0.1 (0.0-0.2) [54]
Ferritin (ng/mL)	1326.8 (732-2180) [98]	1115.8 (1219.1-3878.2) [23]	1152.8 (555-2131.2) [75]
Leukocyte count (/µL)	9260 (6520-11990) [101]	10030 (6980-16320) [25]	9195 (6510-11865) [76]
Neutrophil count (/µL)	7530 (5400-10275) [100]	8605 (5795-15240) [24]	7435 (5230-9590) [76]
Lymphocyte count (/µL)	693.5 (479.5-1045) [100]	605 (370-870) [24]	725 (500-1215) [76]
Platelet count (/µL)	293500 (230500-381500) [100]	244000 (189500-282500) [24]	36500 (248500-411000) [76]
IL-6 (pg/mL)	119.2 (42.3-700.6) [31]	1293.2 (892.1-1500) [6]	77.7 (29.2-276) [25]
DD (mg/L)	1.7 (0.7-4.4) [100]	2.3 (1.3-5.1) [25]	1.4 (0.6-4.0) [75]
Fibrinogen (mg/dL), mean (SD)	451.3 (185.3) [72]	454.2 (189.6) [20]	450.2 (185.5) [52]
PaO_2/FiO_2 (mmHg)	200 (172-286) [107]	191 (160-220) [28]	207 (176-289) [79]
Temperature (ºC), mean (SD)	36.1 (0.9) [42]	36.5 (1.7) [9]	36 (0.5) [33]
**Laboratory/clinical findings** **6 days post-TCZ,** median (IQR) [N] except when indicated			
Total serum proteins (gr/dL), mean (SD)	5.9 (0.6) [79]	5.9 (0.7) [19]	6.0 (0.6) [60]
Albumin (gr/dL)	3.2 (3.0-3.5) [68]	3.1 (2.9-3.3) [19]	3.3 (3.0-3.6) [49]
AST (U/L)	32.5 (23.0-50.0) [88]	32.5 (23.0-57.0) [20]	32.5 (23.0-48.5) [68]
ALT (U/L)	62.0 (37.0-118.0) [90]	46.0 (26.0-100.0) [21]	64 (42-118) [69]
GGT (U/L)	121 (68-291) [67]	101.5 (58-302) [18]	121 (73-277) [49]
LDH (U/L)	401 (322-496) [90]	572 (425-603) [21]	383 (317-439) [69]
Troponin I (pg/mL)	4.7 (2.3-18.3) [55]	19.1 (8.6-134.5) [15]	4 (1.9-6.8) [40]
CRP (mg/L)	4.9 (2.2-9.2) [89]	7.5 (3.7-13.5) [20]	4.3 (2.0-8.8) [69]
Procalcitonine (ng/mL)	0.1 (0.0-0.1) [61]	0.1 (0.1-0.3) [17]	0.0 (0.0-0.1) [44]
Ferritin (ng/mL)	1288.4 (840-2134.1) [85]	1494 (1090-3469.3) [19]	1278 (728.5-1905) [66]
Leukocyte count (/µL)	11490 (8780-14490) [91]	14545 (9120-23570) [22]	11340 (8690-13620) [69]
Neutrophil count (/µL)	8915 (7070-13122) [90]	12020 (7710-15830) [21]	8340 (6960-12520) [69]
Lymphocyte count (/µL)	950 (610-1670) [90]	730 (600-920) [21]	1040 (620-1820) [69]
Platelet count (/µL)	302000 (231000-378000) [90]	213000 (189000-296000) [21]	322000 (247000-432000) [69]
IL-6 (pg/mL)	137.9 (31-566) [22]	645.4 (520-3206) [6]	66 (6.3-273.2) [16]
DD (mg/L)	2.2 (0.8-5.5) [87]	5.3 (1.4-18.9) [21]	2 (0.7-4.7) [76]
Fibrinogen (mg/dL), mean (SD)	323.2 (148.4) [63]	314.4 (156.8) [17]	326.4 (146.8) [46]
PaO_2/FiO_2 (mmHg)	226 (180-325) [96]	185 (180-230) [21]	250 (182-355) [75]
Temperature (ºC), mean (SD)	36.2 (0.7) [40]	36.5 (1.5) [5]	36.1 (0.6) [35]

SD: Standard deviation; IQR: interquartile range; AST: Aspartate aminotransferase; ALT: Alanine aminotransferase; GGT: γ-glutamyl transferase; LDH: Lactate DesHydrogenase; CRP: C- Reactive Protein; IL-6: Interleukin 6; DD: D- Dimer.

Supplementary Table 1 (cont.). Description of analytical parameters measured at day 3, 6 and 9 of patients diagnosed of COVID-19 and receiving tocilizumab (TCZ).

**Laboratory/clinical findings** **9 days post-TCZ** median (IQR) [N] except when indicated	**Total**	**Deceased**	**Survivors**
Total serum proteins (gr/dL), mean (SD)	5.9 (0.7) [67]	5.6 (0.9) [17]	6.0 (0.5) [50]
Albumin (gr/dL)	3.3 (3-3.5) [59]	3.1 (2.9-3.3) [17]	3.3 (3-3.5) [42]
AST (U/L)	30 (22-48) [74]	37 (31-67) [17]	27 (21-45) [57]
ALT (U/L)	65 (44-121) [75]	72 (42-157) [17]	64 (45-115) [58]
GGT (U/L)	152 (67-303) [57]	156 (73-344) [17]	138.5 (66-298.5) [40]
LDH (U/L)	374 (302.5-487.5) [76]	619 (441-794) [17]	361 (279-452) [59]
Troponin I (pg/mL)	10 (3.8-68.7) [42]	74.4 (10.6-389.4) [13]	6.4 (2.9-11.2) [29]
CRP (mg/L)	2.2 (1.2-6.1) [74]	7.8 (2.8-23.6) [16]	1.9 (1.1-4.0) [58]
Procalcitonine (ng/mL)	0.1 (0.0-0.1) [50]	0.2 (0.1-1.5) [16]	0.0 (0.0-0.1) [34]
Ferritin (ng/mL)	1339.9 (578.1-2088.2) [74]	1567 (1058.7-4043.5) [17]	1165 (490.2-1837.3) [57]
Leukocyte count (/µL)	11830 (8255-16825) [76]	18570 (14540-26530) [17]	9920 (7720-14040) [59]
Neutrophil count (/µL)	8485 (5830-14665) [76]	15880 (12900-22240) [17]	7730 (5080-11050) [59]
Lymphocyte count (/µL)	1240 (680-1810) [75]	770 (565-995) [16]	1340 (860-1960) [59]
Platelet count (/µL)	264500 (179000-321500) [76]	207000 (155000-274000) [17]	281000 (206000-338000) [59]
IL-6 (pg/mL)	33 (9.8-40.7) [5]	N/A [0]	33 (9.8-40.7) [5]
DD (mg/L)	1.7 (0.9-3.9) [75]	3.2 (1.2-7.3) [17]	1.5 (0.8-3.4) [58]
Fibrinogen (mg/dL), mean (SD)	318.9 (165.5) [53]	336.5 (131.3) [16]	311.4 (179.3) [37]
PaO_2/FiO_2 (mmHg)	245.5 (171-354.5) [76]	174 (160-222) [18]	285.5 (190-380) [31]
Temperature (ºC), mean (SD)	36.2 (0.8) [23]	38 (0) [2]	36.0 (0.6) [21]

SD: Standard deviation; IQR: interquartile range; AST: Aspartate aminotransferase; ALT: Alanine aminotransferase; GGT: γ-glutamyl transferase; LDH: Lactate DesHydrogenase; CRP: C- Reactive Protein; IL-6: Interleukin 6; DD: D- Dimer.
